# Association between erectile dysfunction and the predicted 10-year risk for atherosclerosis cardiovascular disease among U.S. men: a population-based study from the NHANES 2001-2004

**DOI:** 10.3389/fendo.2024.1442904

**Published:** 2024-12-17

**Authors:** Yangyang Mei, Yiming Chen, Xiaogang Wang, Renfang Xu, Rui Xu, Xingliang Feng

**Affiliations:** ^1^ Department of Urology, Jiangyin People’s Hospital Affiliated to Nantong University, Jiangyin, Jiangsu, China; ^2^ Department of Urology, The Third Affiliated Hospital of Soochow University, Changzhou, Jiangsu, China; ^3^ Department of Urology, The First People’s Hospital of Changzhou, Changzhou, Jiangsu, China; ^4^ Department of Rehabilitation Medicine, Affiliated Jinhua Hospital, Zhejiang University School of Medicine, Jinhua Municipal Central Hospital, Jinhua, Zhejiang, China

**Keywords:** erectile dysfunction, the 10-year atherosclerotic cardiovascular disease, cardiovascular health, NHANES, reciprocal association

## Abstract

**Background:**

Erectile dysfunction (ED) is considered the tip of the iceberg for cardiovascular disease (CVD). However, there is still conflicting evidence regarding their relationship. Recently, a validated tool for the Atherosclerotic Cardiovascular Disease (ASCVD) risk score has provided a key opportunity to delve deeper into the relationship between ED and CVD. Therefore, we intended to assess the relationship between ED and 10-year ASCVD risk score

**Methods:**

Complete data of 1207 participants from the 2001-2004 National Health and Nutrition Examination Survey (NHANES) were used in the study. Various weighted logistic and linear regression models were employed to investigate the effect of the presence of ED on the higher 10-Year ASCVD risk score or high risk of 10-Year ASCVD. Conversely, logistic regression models were repeated to explore the effect of continuous or categorical ASCVD risk score on the prevalence of ED. Sensitivity analyses were also conducted, focusing on severe ED with a more stringent definition. Additionally, we supplemented our study with subgroup analyses, restricted cubic spline (RCS) analysis, and receiver operating characteristic (ROC) analysis to enhance the robustness of our results.

**Results:**

Participants with ED had higher ASCVD risk scores and a higher risk of ASCVD, which corresponded to a greater prevalence of ED or severe ED. When considering the presence of ED as the exposure, our results indicated that the presence of ED increased the ASCVD risk score (Model 3: β [95%CI]: 2.09 [1.12, 3.06]) in Model 3, as well as the high risk of ASCVD (OR [95%CI]: 2.27 [1.13, 4.59]). Conversely, a continuous increase in the ASCVD risk score was also associated with an increased prevalence of ED (OR [95%CI]: 1.04 [1.02,1.06]). Additionally, those in the borderline ASCVD risk group (OR [95% CI]: 2.95 [1.60, 5.44]), intermediate ASCVD risk group (OR [95% CI]: 4.53 [2.35, 8.73]), and high ASCVD risk group (OR [95% CI]: 7.62 [3.19, 18.19]) exhibited progressively increasing ED risk when compared to the low-risk group. Furthermore, the RCS analysis demonstrated a linear relationship between ED prevalence and the continuous ASCVD risk score, with the latter showing high efficacy in predicting ED (AUC [95%CI]: 0.794 [0.768, 0.821]).

**Conclusions:**

The presence of ED may precede the onset of ASCVD by some years. Consequently, timely and dynamic evaluation of the cardiovascular status provides an earlier opportunity to identify and implement effective prevention strategies to promote cardiovascular health for ED patients.

## 1 Introduction

Erectile dysfunction (ED), defined as the persistent or recurrent inability to achieve and/or maintain a penile erection sufficient for satisfactory sexual performance (1), is a prevalent complaint among middle-aged and elderly men. Recent studies reveal a rising prevalence, with ED affecting 14.8% of men aged 40-59, 43.8% of men aged 60-69, and 70% of men over 70, all of which are substantially higher than the 5.1% observed in men under 40 (2, 3). Currently, ED affects approximately 150 million men worldwide, with this number expected to reach 300 million by 2025, largely due to population aging (4). This widespread condition not only diminishes sexual health and quality of life but also imposes a significant economic burden (5). Additionally, the aging population has contributed to an increased burden of cardiovascular diseases (CVD), further exacerbating the public health challenge.

ED has gained attention for its role as both a sentinel symptom and an independent risk factor for CVD, which are the leading causes of morbidity and mortality globally (6). However, traditional CVD risk evaluations fail to identify a significant portion of seemingly healthy men who are at risk for future CVD (7). ED has garnered increasing attention from researchers because it is now recognized as both a sentinel symptom of subclinical CVD and an independent cardiovascular risk factor (8). Mechanistically, the artery size hypothesis explains the link between ED and CVD by proposing that smaller penile arteries are more susceptible to systemic risk factors than larger vessels in the heart, causing the same level of plaque buildup to compromise blood flow more profoundly in the penile arteries and making the resulting decrease in erectile rigidity more noticeable than symptoms from larger cardiovascular blockages (9, 10). However, conflicting results have emerged regarding the independent association between ED and CVD mortality, underscoring the need for further investigation (11). Such robust evidences could increase awareness of ED, making ED consultations a critical opportunity for identifying and promptly intervening in CVD (12).

Recently, a validated tool to predict an individual’s 10-year risk of developing CVD has been developed by the American College of Cardiology (ACC) and the American Heart Association (AHA), known as the Atherosclerotic Cardiovascular Disease (ASCVD) risk score (13, 14). This tool provides new opportunities to verify the relationship between ED and CVD risk without requiring long-term follow-up. Additionally, the NHANES database provides a unique platform for population-based studies to explore this association. Consequently, the present study was conducted to comprehensively explore the bidirectional association between ED and CVD. The objectives of this study are twofold: first, to determine whether ED increases the 10-year ASCVD risk score, thus placing patients at high risk of ASCVD; and second, to assess the relationship between the 10-year ASCVD risk score and ED, including evaluating whether there is a linear relationship and the effectiveness of using the 10-year ASCVD risk score to predict ED. In our study, we hypothesize a bidirectional relationship where ED can elevate the 10-year ASCVD risk score, and conversely, the 10-year ASCVD risk score can predict ED, highlighting the importance of early intervention and prevention of CVD events in ED patients.

## 2 Materials and methods

### 2.1 Data source and study population

Data from the 2001-2004 NHANES were used in the study, as ED evaluation and 10-year ASCVD risk scores were only available in these cycles. NHANES is a continuous biennial cross-sectional study by the National Center for Health Statistics (NCHS) of the Centers for Disease Control and Prevention (CDC), designed to assess the health and nutritional status of the U.S. population. To achieve a representative sample of the U.S. population, the study employs a stratified, multistage probability cluster sampling design. NHANES data comprises five components: sociodemographic characteristics, physical examinations, dietary data, laboratory tests, and health status, all gathered by experienced medical personnel through interviews and physical exams to ensure data accuracy and professionalism. The NCHS Research Ethics Committee (Approval No. Protocol #98-12) approved all protocols and procedures, and all participants provided written informed consent before participation.

**Figure 1:**
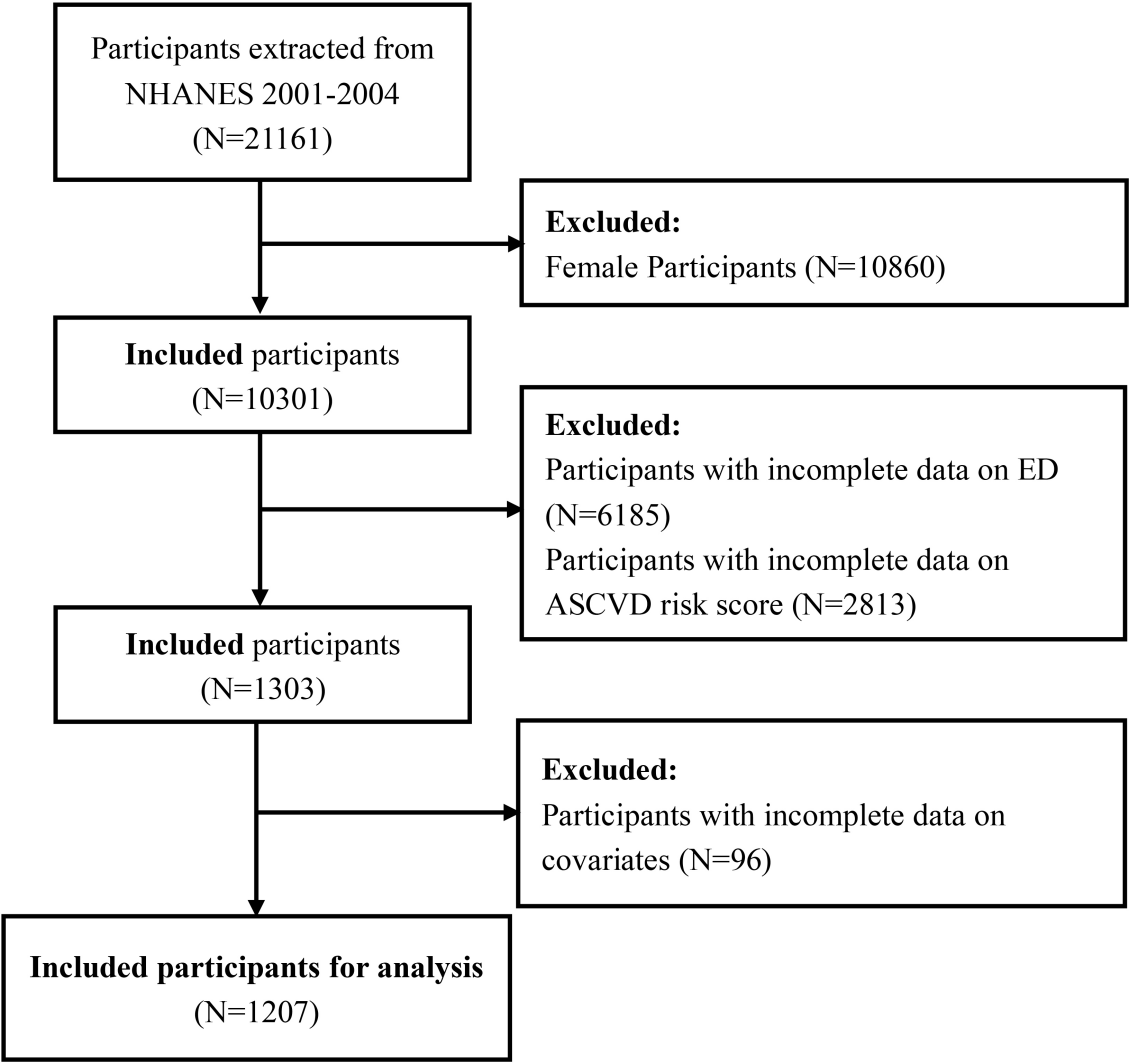
Flowchart of study population selection process and exclusion criteria.

Initially, a total of 21,161 participants were involved in NHANES 2001-2004. Based on the study objectives, a series of exclusion criteria were established to determine the final study population. First, 10,860 female participants were excluded. Next, 6,185 male participants without ED data were excluded, including those under 20 years of age and those who did not complete the ED questionnaire. Subsequently, 2,813 participants without ASCVD risk score data were excluded, narrowing the study population to those who (1) were aged 40-79 years; (2) were of Non-Hispanic Whites; and (3) had available data on smoking, arterial hypertension, hypertension treatment, cholesterol levels, and diabetes mellitus (DM). Additionally, 96 participants with missing covariate data were also excluded. Ultimately, 1,207 participants met the inclusion criteria for the final analysis, comprising 418 participants with ED and 789 controls without ED. The detailed selection process is illustrated in **Figure 1**.

### 2.2 Evaluation of the exposure of interest: erectile dysfunction

The exposure of interest in the study is the history of ED, assessed by trained interviewers asking participants the question: “How would you describe your ability to get and keep an erection adequate for satisfactory intercourse? (Prostate Conditions- KIQ400)”. This straightforward question was developed by the Massachusetts Male Aging Study (MMAS) and has been validated for reliability (15). The answers included “always or almost always able,” “usually able,” “sometimes able,” and “never able.” Consistent with previous studies (16, 17), participants who answered “always or almost always able” or “usually able” were classified as not having ED, while the remaining participants were classified as having ED. It is important to note that only men aged 20 and older in the 2001-2004 cycles received this survey question.

### 2.3 Evaluation of the outcome of interest: 10-year ASCVD risk

The outcome of interest in the study was the 10-year ASCVD risk, defined as the first occurrence of non-fatal myocardial infarction (MI), coronary heart disease (CHD) death, or fatal/non-fatal stroke within a decade (18). However, the 10-year ASCVD risk is currently applicable only to non-Hispanic whites aged 40–79 years, according to the 2013 ACC/AHA recommendations (18). The pooled cohort equations for estimating 10-year ASCVD risk incorporate eight predictors: age, sex, race, total cholesterol (TC), high-density lipoprotein cholesterol (HDL-C), arterial pressure, hypertension treatment, diabetes, and current smoking status. Among these predictors, age, sex, and race data were obtained from the demographic questionnaire, while information on smoking, hypertension medication use, and diabetes was gathered through health status questionnaires. Additionally, blood pressure was measured during the physical examination as the mean of three readings, while TC (mg/dL) and HDL (mg/dL) were obtained from laboratory tests. Drawing from prior research, participants with a 10-year ASCVD risk score of ≥7.5% were classified as high-risk, while those with a score <7.5% were considered low-risk (19). In our secondary analysis of the relationship between the 10-year ASCVD risk score and ED, the 10-year ASCVD risk score was treated as the exposure and used as a continuous variable when ED was the outcome. Additionally, we categorized the 10-year ASCVD risk score into multiple groups according to guidelines: <5.0% (low risk), 5.0% to <7.5% (borderline risk), 7.5% to <20.0% (intermediate risk), and ≥20.0% (high risk) (13).

### 2.4 Evaluation of potential covariates

Based on existing experience and previous studies, potential covariates that may influence the relationship between ED and 10-year ASCVD risk include age, body mass index (BMI), education level, marital status, poverty-income ratio (PIR), alcohol intake, smoking status, physical activity (vigorous or moderate), and history of DM, hypertension, hyperlipidemia and CVD. Age was categorized into two categories: <60 years and ≥60 years. BMI was categorized into three groups: <25 kg/m^2^, 25-30 kg/m^2^, and ≥30 kg/m^2^. The PIR was categorized into three groups: <1.3, 1.3-3.5, and ≥3.5. Marital status was classified as either married or living with a partner or living alone, while educational level was divided into less than high school, high school or equivalent, and above high school or college. Participants were classified as drinkers if they consumed more than one alcoholic drink per day on average; otherwise, they were considered non-drinkers. Regarding smoking status, participants were classified as current smokers if they had smoked >100 cigarettes in their lifetime and currently smoked daily or occasionally. Those who had never smoked >100 cigarettes in their lifetime were considered non-smokers, and the remainder were classified as former smokers. Physical activity status (moderate or vigorous) was determined based on responses to a single survey question about participation in moderate or vigorous activity during the past month.

DM was considered present if participants met any of the following criteria: (1) a previous diagnosis of DM by a doctor; (2) current use of antidiabetic medications or insulin; (3) laboratory results meeting any of the following conditions: glycosylated hemoglobin (HbA1c) ≥ 6.5%, fasting blood glucose ≥ 126 mg/dL, a plasma glucose level ≥ 200 mg/dL at 2 hours after an oral glucose tolerance test (OGTT). Similarly, hypertension was considered present if participants met any of the following criteria: (1) a self-reported diagnosis of hypertension; (2) current use of antihypertensive medications; or (3) examination results showing a diastolic blood pressure ≥ 90 mm Hg or a systolic blood pressure ≥ 140 mm Hg. Hyperlipidemia was considered present if participants met any of the following criteria: (1) a previous diagnosis of high cholesterol; (2) the prescription of cholesterol-lowering medication; or (3) a total serum cholesterol level ≥240 mg/dL. In contrast, a diagnosis of CVD was based solely on self-reported questionnaire responses indicating a history of angina, heart attack, or coronary heart disease.

### 2.5 Statistical analysis

Considering the complex multistage cluster survey design of NHANES, all statistical analyses applied appropriate sample weights and strictly adhered to CDC guidelines for NHANES data analysis. Based on the study design, appropriate Mobile Examination Center (MEC) weights were utilized to obtain prevalence estimates representative of the US population. Given the combination of the two cross-sectional studies, the original MEC weights were multiplied by 0.5 to derive the final weights. In descriptive analyses, continuous variables were presented as weighted mean ± standard error (SE), while categorical variables were expressed as weighted percentages with 95% confidence intervals (CIs). Thereafter, the chi-square test for categorical variables and analysis of variance (ANOVA) for continuous variables were used to assess differences between those with and without a history of ED and among groups with different 10-year ASCVD risk levels. To investigate the relationship between 10-year ASCVD risk and ED, various logistic and linear regression models, were employed. Both linear and logistic regression models adjusted for covariates consistently, except for the exposure and outcome variables. Model 1, also known as the crude model, had no additional variables adjusted. Model 2, the minimally adjusted model, adjusted for age, education, marital status, and PIR. Model 3, the fully adjusted model, included all adjustments from Model 2 plus following variables: BMI, hypertension, DM, CVD, hyperlipidemia, alcohol consumption, smoking status, vigorous activity, and moderate activity.

First, we used linear regression to treat the 10-year ASCVD risk as a continuous variable (reported as β with 95% CI) and logistic regression for the binary outcome (with a 7.5% threshold, reported as OR with 95% CI) to examine the relationship between ED and 10-year ASCVD risk, with ED as the exposure variable. Next, logistic regression was applied to explore the association between the 10-year ASCVD risk score and ED prevalence, treating the risk score both as a continuous and categorical variable, with ED as the outcome variable. To enhance the robustness of our findings, we stratified participants by age, BMI, smoking status, hypertension, DM, and CVD, and performed interaction analyses to investigate potential differential associations among subgroups. Additionally, sensitivity analyses were performed by focusing on participants with severe ED, including only those who responded “never able” to the KIQ400 questionnaire.

Moreover, when the 10-year ASCVD risk score was considered a continuous exposure variable, weighted restricted cubic splines (RCSs) were used to elucidate the dose-response relationship between the 10-year ASCVD risk score and ED prevalence. Receiver operating characteristic (ROC) curves were further used to evaluate the predictive ability of the 10-year ASCVD risk score for ED by calculating the corresponding areas under the curve (AUCs) and their 95% CIs. A bilateral P-value of <0.05 was considered statistically significant in all analyses. All statistical analyses were conducted using EmpowerStats software (www.empowerstats.com; X&Y Solutions, Inc., Boston, MA) and R software (R 4.2.3; http://www.R-project.org; The R Foundation, Vienna, Austria).

## 3 Results

### 3.1 Baseline characteristics of study population

The baseline characteristics of participants with and without a history of ED are presented in **Table 1**. Compared to participants without ED, those with ED exhibited older age (63.29 ± 0.42 years vs 51.21 ± 0.27 years), higher BMI (29.74 ± 0.41 kg/m^2^ vs 28.45 ± 0.24 kg/m^2^), and higher ASCVD risk scores (19.93% ± 0.59 vs 8.15% ± 0.28). Additionally, other variables also exhibited significant differences between the groups, including education level, PIR, alcohol intake, smoking status, vigorous activity, and history of DM, hypertension, hyperlipidemia and CVD. Notably, participants with ED exhibited a higher proportion of intermediate (40.47% vs 26.99%) and high 10-year ASCVD risk (41.00% vs 8.68%). Similarly, participants were grouped according to their 10-year ASCVD risk, with the detailed results presented in **Supplementary Table S1**.

**Table 1:** Baseline characteristics of participants with or without erectile dysfunction in NHANES 2001-2004, Weighted.

Characteristics	Total participants	History of erectile dysfunction (ED)		P value
		No	Yes	
Number, n	1207	789	418	
Age, year	54.35 ± 0.27	51.21 ± 0.27	63.29 ± 0.42	< 0.0001
BMI, kg/m^2^	28.79 ± 0.19	28.45 ± 0.24	29.74 ± 0.41	0.01
TC, mg/dL	207.53 ± 1.63	209.62 ± 1.95	201.56 ± 3.20	0.04
HDL-c, mg/dL	46.63 ± 0.46	46.88 ± 0.57	45.93 ± 0.51	0.19
ASCVD risk score, %	11.21 ± 0.32	8.15 ± 0.28	19.93 ± 0.59	< 0.0001
Educational level, %				< 0.0001
Below high school	10.62 (7.74, 13.49)	7.15 (5.03, 9.27)	20.49 (15.70, 25.27)	
High school	27.43 (21.98, 32.88)	28.26 (24.79, 31.72)	25.08 (21.26, 28.90)	
Above high school	61.95 (54.07, 69.84)	64.60 (60.49, 68.70)	54.43 (49.18, 59.68)	
Marital status, %				0.33
Married or living with a partner	81.21 (68.91,93.51)	80.58 (77.39,83.77)	83.00 (79.42,86.58)	
Living alone	18.79 (15.95,21.63)	19.42 (16.23,22.61)	17.00 (13.42,20.58)	
PIR, %				< 0.001
PIR ≤ 1.3	10.36 (7.14, 13.59)	9.69 (7.49, 11.89)	12.29 (7.39, 17.18)	
1.3<PIR ≤ 3.5	29.77 (24.85, 34.69)	26.77 (23.29, 30.24)	38.33 (33.33, 43.34)	
PIR>3.5	59.86 (51.21, 68.52)	63.54 (59.18, 67.91)	49.38 (42.24, 56.52)	
BMI category, %				0.03
<25 kg/m^2^	21.17 (16.90,25.44)	21.87 (18.17,25.57)	19.17 (14.63,23.71)	
25-30 kg/m^2^	45.13 (38.67,51.58)	47.01 (43.68,50.35)	39.75 (35.58,43.92)	
>=30 kg/m^2^	33.71 (28.39,39.03)	31.12 (27.36,34.88)	41.08 (35.24,46.92)	
Age category, %				< 0.0001
<60y	70.15 (61.11,79.18)	81.69 (79.75,83.64)	37.27 (32.98,41.56)	
≥60y	29.85 (24.86,34.84)	18.31 (16.36,20.25)	62.73 (58.44,67.02)	
Alcohol intake, %				< 0.0001
No	27.04 (20.39,33.68)	22.99 (17.76,28.23)	38.55 (32.38,44.71)	
Yes	72.96 (62.82,83.11)	77.01 (71.77,82.24)	61.45 (55.29,67.62)	
Smoking, %				< 0.0001
Never	38.55 (33.08,44.02)	41.84 (37.57,46.11)	29.20 (23.60,34.79)	
Former	39.33 (32.91,45.76)	34.96 (31.78,38.15)	51.78 (45.63,57.94)	
Now	22.11 (17.76,26.47)	23.20 (20.77,25.62)	19.02 (13.46,24.58)	
Vigorous activity, %				< 0.0001
No	66.23 (56.84, 75.62)	61.27 (57.47, 65.06)	80.36 (76.33, 84.39)	
Yes	33.77 (27.86, 39.68)	38.73 (34.94, 42.53)	19.64 (15.61, 23.67)	
Moderate activity, %				0.09
No	41.19 (35.41, 46.97)	39.62 (35.96, 43.27)	45.66 (40.10, 51.22)	
Yes	58.81 (49.67, 67.95)	60.38 (56.73, 64.04)	54.34 (48.78, 59.90)	
History of DM, %				< 0.0001
No	87.03 (75.32, 98.73)	92.76 (90.88, 94.63)	70.72 (66.42, 75.01)	
Yes	12.97 (9.97, 15.97)	7.24 (5.37, 9.12)	29.28 (24.99, 33.58)	
History of CVD, %				< 0.0001
No	92.24 (79.88, 104.61)	95.18 (93.59, 96.77)	83.89 (79.61, 88.17)	
Yes	7.76 (5.53, 9.98)	4.82 (3.23, 6.41)	16.11 (11.83, 20.39)	
History of hypertension, %				< 0.0001
No	56.62 (47.63, 65.62)	62.53 (57.44, 67.62)	39.80 (35.74, 43.87)	
Yes	43.38 (36.32, 50.43)	37.47 (32.38, 42.56)	60.20 (56.13, 64.26)	
History of hyperlipidemia, %				0.40
No	17.57 (14.10, 21.04)	18.08 (15.63, 20.53)	16.12 (11.90, 20.34)	
Yes	82.43 (71.15, 93.71)	81.92 (79.47, 84.37)	83.88 (79.66, 88.10)	
ASCVD risk factor category, %				< 0.0001
<5%	39.40 (33.85,44.95)	49.83 (45.78, 53.88)	9.70 (6.21, 13.18)	
5%-7.5%	13.02 (10.43,15.62)	14.50 (11.72, 17.27)	8.83 (5.64, 12.03)	
7.5%-20%	30.50 (24.38,36.61)	26.99 (24.01, 29.98)	40.47 (35.87, 45.07)	
>20%	17.08 (14.19,19.97)	8.68 (6.95, 10.40)	41.00 (37.35, 44.66)	

ED, erectile dysfunction; BMI, body mass index; PIR, poverty income ratio; TC, total cholesterol; HDL-c, high-density lipoprotein cholesterol; ASCVD, atherosclerotic cardiovascular disease; DM, diabetes mellitus; CVD, cardiovascular disease.

### 3.2 Reciprocal relationship between ED and 10-Year ASCVD risk

**Table 2** displays the results of the linear and logistic regression analyses, with ED as the exposure variable and 10-Year ASCVD Risk as the outcome variable. The results show that the presence of ED increased the 10-Year ASCVD Risk score across all models (Model 3: β [95%CI]: 2.09 [1.12, 3.06]). Furthermore, it also significantly increased the 10-Year ASCVD risk for patients (Model 3: OR [95%CI]: 2.27 [1.13, 4.59]). Next, the results of the logistic regression analysis are shown in **Supplementary Table S2**, with ED as the outcome variable. In the fully adjusted Model 3, each unit increase in the 10-Year ASCVD risk score was associated with a 4% increase in ED risk (OR [95%CI]: 1.04 [1.02,1.06]). Compared with participants in the low 10-Year ASCVD risk group, those in the borderline10-Year ASCVD risk (OR [95%CI]: 2.95 [1.60, 5.44]), intermediate 10-Year ASCVD risk (OR [95%CI]: 4.53 [2.35, 8.73]), and high 10-Year ASCVD risk (OR [95%CI]: 7.62 [3.19,18.19]) groups exhibited progressively increasing ED risk, with a trend test p-value < 0.05.

**Table 2:** Multivariable logistic regression analyses for ED and 10-year ASCVD risk score or high risk of 10-year ASCVD, weighted.

Erectile dysfunction	Adjusted Model 1		Adjusted Model 2		Adjusted Model 3	
10-year ASCVD score-β (95%CI)-P value						
No	Ref	Ref	Ref	Ref	Ref	Ref
Yes	11.79 (10.51,13.06)	<0.0001	4.33 (3.40, 5.26)	<0.0001	2.09 (1.12, 3.06)	0.001
10-year high risk of ASCVD-OR (95%CI) -P value						
No	Ref	Ref	Ref	Ref	Ref	Ref
Yes	7.93 (5.56,11.30)	<0.0001	3.55 (2.25, 5.60)	<0.0001	2.27 (1.13, 4.59)	0.027

ED, erectile dysfunction; BMI, body mass index; PIR, poverty income ratio; ASCVD, atherosclerotic cardiovascular disease; DM, diabetes mellitus; CVD, cardiovascular disease; OR, odds ratios; 95%CI, 95% confidence intervals.

Model 1: unadjusted.

Model 2: adjusted for age, education level, marital status, and PIR.

Model 3: age, education level, marital status, PIR, BMI, hypertension, DM, CVD, hyperlipidemia, alcohol consumption, smoking status, vigorous activity, and moderate activity.

β, effect size for linear regression.

### 3.3 Sensitivity analysis

All the aforementioned regression analyses were repeated during the sensitivity analysis. As shown in **Table 3**, the results indicate that severe ED increases both the 10-Year ASCVD risk score (β [95%CI]: 1.56 [0.88, 2.25]) and the 10-Year ASCVD risk (OR [95%CI]: 1.62 [1.05, 2.49]) when all potential covariates are adjusted. **Supplementary Table S3** shows that a higher 10-Year ASCVD risk score is associated with a higher prevalence of severe ED. In Model 3, each unit increase in the 10-Year ASCVD risk score was associated with a 7% increase in severe ED [OR (95%CI): 1.07(1.04,1.10)]. Similarly, participants in the borderline 10-Year ASCVD risk group (OR [95% CI]: 1.73 [1.19,2.51]), intermediate 10-Year ASCVD risk group (OR [95% CI]: 2.01 [1.27,3.20]), and high 10-Year ASCVD risk group (Model 3: OR [95% CI]: 4.11 [1.97,8.54]) exhibited progressively increasing ED risk compared to those in the low 10-Year ASCVD risk group. Surely, the trend test continued to show statistical significance.

**Table 3:** Sensitivity analysis for ED and 10-year ASCVD risk score or high risk of 10-year ASCVD, weighted.

Erectile dysfunction	Adjusted Model 1		Adjusted Model 2		Adjusted Model 3	
10-year ASCVD score-β (95%CI)-P value						
No	Ref	Ref	Ref	Ref	Ref	Ref
Severe ED	10.0 (9.08,10.92)	<0.0001	3.51 (2.61, 4.42)	<0.0001	1.56 (0.88, 2.25)	<0.001
10-year high risk of ASCVD-OR (95%CI) -P value						
No	Ref	Ref	Ref	Ref	Ref	Ref
Severe ED	5.49 (4.38,6.88)	<0.0001	2.49 (1.80, 3.44)	<0.0001	1.62 (1.05, 2.49)	0.032

ED, erectile dysfunction; BMI, body mass index; PIR, poverty income ratio; ASCVD, atherosclerotic cardiovascular disease; DM, diabetes mellitus; CVD, cardiovascular disease; OR, odds ratios; 95%CI, 95% confidence intervals.

Model 1: unadjusted.

Model 2: adjusted for age, education level, marital status, and PIR.

Model 3: age, education level, marital status, PIR, BMI, hypertension, DM, CVD, hyperlipidemia, alcohol consumption, smoking status, vigorous activity, and moderate activity.

β, effect size for linear regression.

### 3.4 Supplementary subgroup Analysis, RCS analysis, and ROC analysis

**Figure 2:**
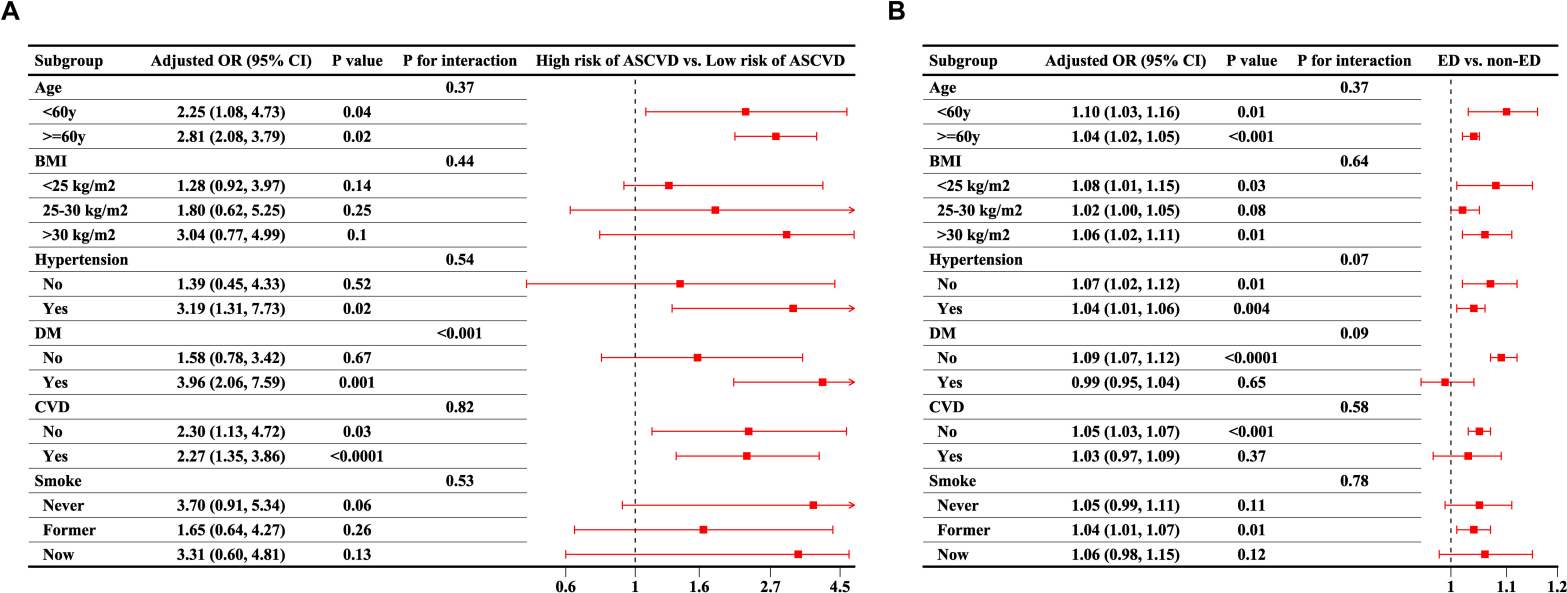
Subgroup analysis for the reciprocal association between ED and 10-year ASCVD risk. **(A)** Association between presence of ED and elevated 10-year ASCVD risk; **(B)** Association between 10-year ASCVD risk score and presence of ED. All subgroup analyses were adjusted for age, education level, marital status, PIR, BMI, hypertension, DM, CVD, hyperlipidemia, alcohol consumption, smoking status, vigorous activity, and moderate activity, as included in Model 3, except for the grouping variable. ED, erectile dysfunction; BMI, body mass index; PIR, poverty income ratio; ASCVD, atherosclerotic cardiovascular disease; DM, diabetes mellitus; CVD, cardiovascular disease; OR, odds ratios; 95%CI, 95% confidence intervals.

**Figure 3:**
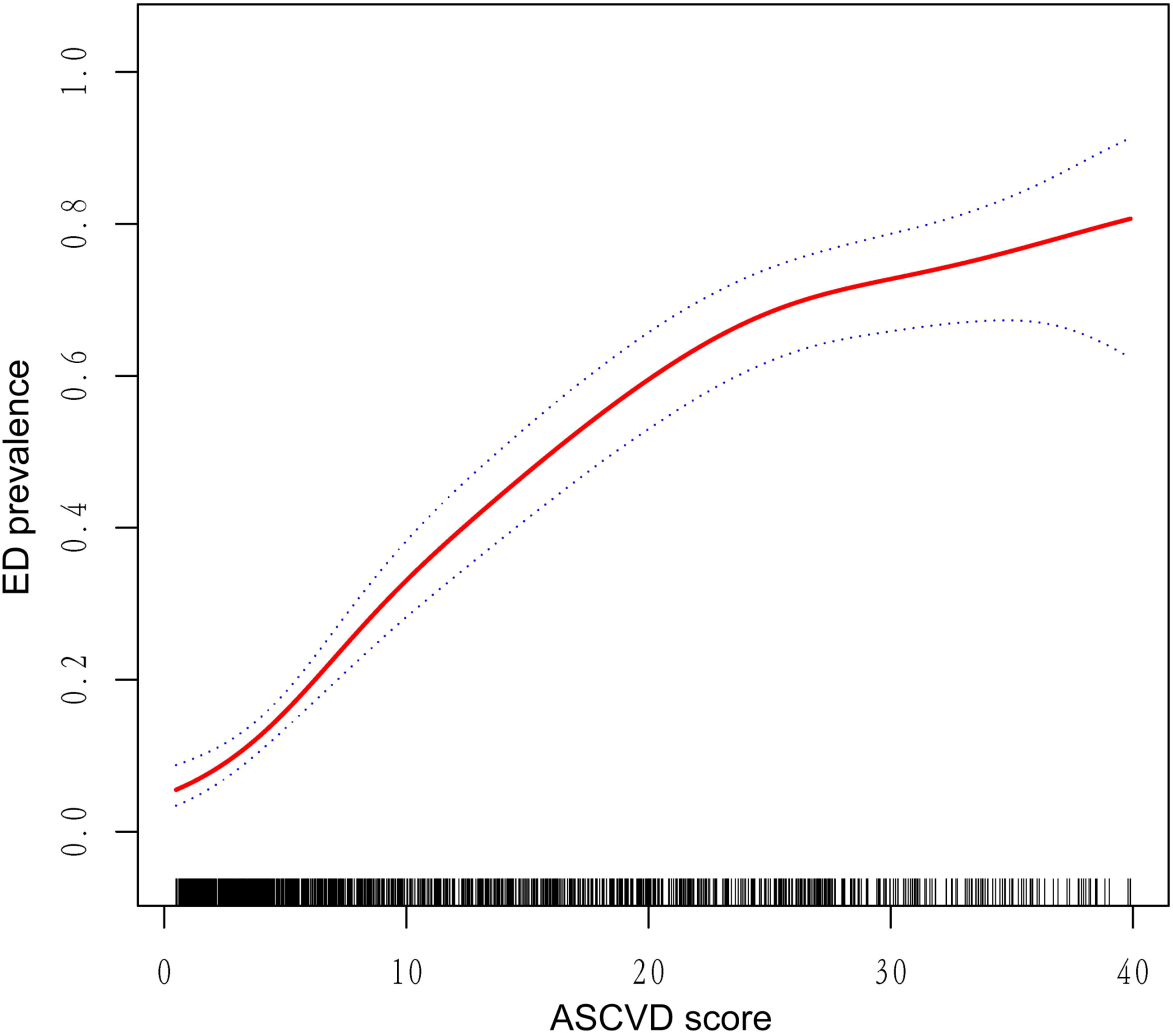
Dose–response relationship analysis between continuous 10-year ASCVD risk score and ED prevalence. RCS regression was adjusted for age, education level, marital status, PIR, BMI, hypertension, DM, CVD, hyperlipidemia, alcohol consumption, smoking status, vigorous activity, and moderate activity (Model 3). The red solid line represents ORs, and black dashed region represents the 95% CI. ED, erectile dysfunction; BMI, body mass index; PIR, poverty income ratio; ASCVD, atherosclerotic cardiovascular disease; DM, diabetes mellitus; CVD, cardiovascular disease; OR, odds ratios; 95%CI, 95% confidence intervals; RCS, restricted cubic spline.

Subsequently, three sets of subgroup analyses corresponding to the regression analysis were conducted based on predefined groupings. Firstly, the results of the subgroup analysis concerning the increase in the 10-Year ASCVD risk score related to ED was illustrated in **Table 4**. In the age subgroup, regardless of whether participants were >60 years, the presence of ED increased the 10-Year ASCVD risk score. Additionally, most other subgroups also showed statistically significant results, except for those without DM and CVD, as well as current smokers. Secondly, as shown in **Figure 2A**, participants with ED present a high risk of a 10-Year ASCVD in the subgroup with hypertension, DM, and CVD. Third, **Figure 2B** shows the third set of subgroup analyses, demonstrating the continuous increase in the 10-Year ASCVD risk score in relation to the increasing prevalence of ED. No significant interactions were detected in any of the subgroups (all P-values >0.05). **Figure 3** shows that the dose-response curve analysis of RCS in model 3 demonstrated an increase in the 10-Year ASCVD risk score with a linear increase in ED prevalence. Using the 10-Year ASCVD risk score to predict the presence of ED demonstrated efficacy, with an AUC value of 0.794 (95% CI: 0.768, 0.821) (**Supplementary Figure S1**).

**Table 4:** Subgroup analysis for the association between ED and 10-year ASCVD risk score, weighted.

Subgroup	β (95%CI)	P value	P for interaction
Age			0.07
<60y	1.53 (0.32, 2.73)	0.02	
≥60y	2.71 (1.39, 4.03)	0.002	
BMI			0.78
<25 kg/m^2^	3.06 (0.35, 5.77)	0.03	
25-30 kg/m^2^	1.31 (-0.10, 2.73)	0.07	
>30 kg/m^2^	2.63 (0.85, 4.41)	0.01	
Hypertension			0.20
No	1.62 (0.37, 2.87)	0.02	
Yes	2.45 (0.82, 4.08)	0.01	
DM			0.27
No	-0.75 (-4.12, 2.62)	0.72	
Yes	3.38 (2.28, 4.47)	<0.0001	
CVD			0.84
No	1.79 (-3.04, 6.61)	0.44	
Yes	2.17 (1.15, 3.20)	0.001	
Smoke			0.11
Never	2.03 (-0.11, 4.18)	0.06	
Former	2.29 (0.70, 3.88)	0.01	
Now	1.98 (-1.22, 5.17)	0.17	

ED, erectile dysfunction; BMI, body mass index; PIR, poverty income ratio; ASCVD, atherosclerotic cardiovascular disease; DM, diabetes mellitus; CVD, cardiovascular disease; 95%CI, 95% confidence intervals.

Analyses were adjusted for age, education level, marital status, PIR, BMI, hypertension, DM, CVD, hyperlipidemia, alcohol consumption, smoking status, vigorous activity, and moderate activity except the stratification factor itself.

β, effect size for linear regression.

## 4 Discussion

This cross-sectional study used two consecutive NHANES datasets from 2001-2002 and 2003-2004 to evaluate the reciprocal relationship between ED and ASCVD. Our results revealed that males with the presence of ED showed an increased 10-year ASCVD risk score and a higher risk of 10-year ASCVD. Similarly, our results also showed that males with a higher 10-year ASCVD risk score had a higher risk of ED or severe ED. Subgroup analyses further fortified the stability of the association across diverse subgroups. Additionally, the 10-year ASCVD risk score was linearly associated with ED prevalence and shown to be an effective predictor of ED.

Several studies have confirmed the existence of common risk factors for ED and CVD, such as DM, obesity, metabolic syndrome, dyslipidemia, smoking, and sedentary lifestyle (20–22). As a result, it is widely accepted that ED is an early manifestation of CVD (23–25). In 2005, Thompson et al. first reported the relationship between ED and CVD (26). During the follow-up period of the study, the hazard ratio for subsequent cardiovascular events associated with ED events was 1.25 (95% CI, 1.02-1.53; P = 0.04). In a study involving 1549 male CVD patients, compared to those without ED, men with ED had 1.6-fold increased odds (16.2% vs. 10.3%) of CVD-related death, myocardial infarction, heart failure, or stroke hospitalization during the follow-up period (median of 53-54 months) (27). A large-scale analysis of over 6300 men participating in the ADVANCE (Action in Diabetes and Vascular Disease) study demonstrated that ED is also a particularly strong predictive factor for CVD in men with diabetes (28). Furthermore, erectile dysfunction (ED) typically precedes angina by 2 to 3 years and cardiovascular events by 3 to 5 years (29). In many men, ED may represent the initial manifestation within the clinical disease spectrum, progressing subsequently to include CVD (30). Therefore, early detection of ED and identification of risk factors for both ED and CVD can enable patients to delay or prevent the occurrence of significant adverse cardiovascular events.

The ASCVD risk score is a validated tool developed by the ACC and AHA to predict an individual’s 10-year risk of developing CVD (13, 14). In more detail, the ACC/AHA Task Force on Practice Guidelines emphasizes a scheme for preliminary classification of individual estimated risk levels. Individuals with a 10-year ASCVD risk below 5% are considered low risk, those within the range of 5% to 7.5% are categorized as borderline risk, while those falling between 7.5% and 20% are classified as moderate risk. Finally, individuals with an equal or greater than 20% risk are categorized as high risk. If an individual is determined to be at high risk based on the ASCVD risk score level, statin therapy is strongly recommended; conversely, low-risk individuals do not need to take statins and need to pay attention to developing healthy lifestyle habits to prevent CVD (18). In this regard, the higher the score, the more severe the endothelial dysfunction and atherosclerosis, and Bertini et al. showed that as ASCVD scores increase, the likelihood of arteriogenic ED also increases (31). They were the first to demonstrate that the 10-year ASCVD risk score can be considered an effective and reliable tool for identifying true arteriogenic ED patients. Similarly, our study found that 10-year ASCVD risk scores were linearly associated with ED prevalence. Therefore, people with high ASCVS risk scores should not only be aware of CVD prevention, but may want to be aware of early onset ED symptoms first.

The 10-year ASCVD risk score represents the risk of developing CVD, and ED and CVD share the same major cardiovascular risk factors and pathophysiologic pathways; therefore, the mechanism of the association between the ASCVD risk score and ED may be similar to the mechanism of the association between ED and CVD. Inflammation, atherosclerosis and endothelial dysfunction induced by common risk factors are potential common pathogenic mechanisms for ED and CVD (32, 33). Exposure to vascular risk factors can lead to endothelial dysfunction, which in turn can lead to atherosclerosis (34). Considering the systemic nature of atherosclerosis, atherosclerosis affects all vessels to a similar degree, with arterial diameter determining the time of symptom onset (35–37). In detail, the penile arteries are much smaller compared to the coronary arteries. Smaller atheromatous plaques may develop in the coronary arteries without symptoms, while plaques of the same size may compromise blood flow when they develop in the penile arteries, thus manifesting ED earlier compared to angina (38). For this reason, ED is also metaphorically described as ‘penile angina’ (39).

Our study has several notable strengths worth mentioning. Firstly, to our knowledge, we are the first to use the NHANES database to explore the reciprocal relationship between ED and ASCVD and to confirm their positive interaction, providing more evidence for Urologists/Andrologist to pay attention to the cardiovascular health of ED patients. Secondly, we validated the use of the 10-year ASCVD risk score to predict ED with high efficacy, enabling cardiologists to promptly address sexual function and improve quality of life in ASCVD patients. Thirdly, appropriate sampling weights were considered during the analysis to mitigate oversampling bias, enhancing the reliability of the results. Of course, several limitations of the study should be considered, requiring caution when interpreting our results. Firstly, our study is inherently cross-sectional, using a tool to predict participants’ future 10-year ASCVD risk, which provides a lower level of evidence compared to cohort studies and randomized controlled trials (RCTs). Secondly, the diagnosis of ED was based on a single self-report measure, which, despite being validated, may still lead to recall bias and social desirability bias. Thirdly, due to the inclusion and exclusion criteria, only a limited sample size was included in the analysis, which may introduce potential selection bias. Lastly, despite our efforts to include potential confounders, unmeasured variables may still influence the relationship, and the diagnostic criteria for included variables have certain limitations. Based on these strengths and limitations, our findings are significant for both urologists and cardiologists, but further well-designed studies are needed to confirm these results.

## 5 Conclusion

In summary, our results confirmed the reciprocal positive relationship between ED and the 10-year ASCVD risk. The presence of ED increases the 10-year ASCVD risk, emphasizing the need for cardiovascular screening and early intervention to reduce cardiovascular events. Conversely, a higher ASCVD risk score indicates a greater likelihood of ED, underscoring the importance of sexual health, particularly erectile function, in cardiovascular patients. Additionally, the ASCVD risk score may serve as an efficient tool for predicting ED, potentially useful in clinical practice. However, the inherent limitations of this study highlight the need for further research with better design to provide more robust evidence on the relationship between ED and CVD.

## Data Availability

The raw data supporting the conclusions of this article will be made available by the authors, without undue reservation.

## Supplementary material

Supplementary Figure 1 ROC curves of 10-year ASCVD risk score for discriminating ED. ED: erectile dysfunction; ROC: receiver operating characteristic; ASCVD: atherosclerotic cardiovascular disease; AUC: area under the curve.
