# Supplementary figures and images for: Association between erectile dysfunction and the predicted 10-year risk for atherosclerosis cardiovascular disease among U.S. men: a population-based study from the NHANES 2001-2004
Source: Front Endocrinol (Lausanne). 2024 Dec 17;15:1442904. doi: 10.3389/fendo.2024.1442904 (PMC11685050; doi:10.3389/fendo.2024.1442904)

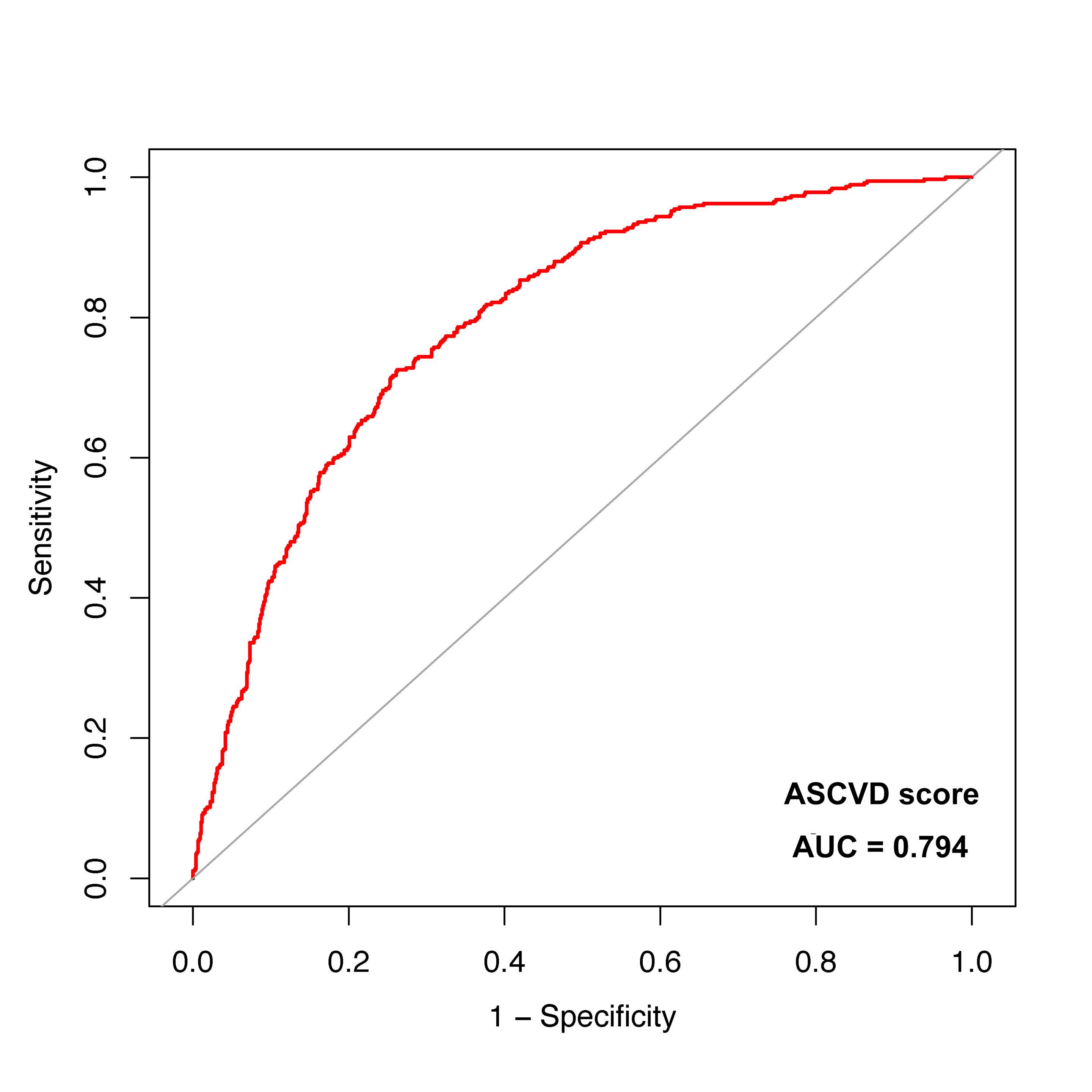

Supplement: Supplementary Figure 1 — ROC curves of 10-year ASCVD risk score for discriminating ED. ED: erectile dysfunction; ROC: receiver operating characteristic; ASCVD: atherosclerotic cardiovascular disease; AUC: area under the curve. [file Image1.jpeg]
