# Supplementary material for: Association between erectile dysfunction and the predicted 10-year risk for atherosclerosis cardiovascular disease among U.S. men: a population-based study from the NHANES 2001-2004
Source: Front Endocrinol (Lausanne). 2024 Dec 17;15:1442904. doi: 10.3389/fendo.2024.1442904 (PMC11685050; doi:10.3389/fendo.2024.1442904)
Supplement: Supplementary file 2 [file Table1.docx]

**Table S1.** Baseline characteristics of participants from 2001-2004 National Health and Nutrition Examination Survey (NHANES), divided by10-year risk of ASCVD, weighted.

| **Characteristics** | Total participants | 10-year risk of ASCVD | | | | **P value** |
| --- | --- | --- | --- | --- | --- | --- |
|  |  | Low (<5%) | Borderline (5%-7.5%) | Intermediate (7.5%-20%) | High (>20%) |  |
| Number, n |  |  |  |  |  |  |
| Age, year | 54.35±0.27 | 45.97±0.23 | 51.25±0.37 | 57.55±0.42 | 70.32±0.53 | < 0.0001 |
| BMI, kg/m^2^ | 28.79±0.19 | 28.00±0.29 | 29.56±0.67 | 29.29±0.39 | 29.11±0.30 | 0.004 |
| TC, mg/dL | 207.53±1.63 | 203.43±2.68 | 209.47±4.08 | 212.57±2.43 | 206.50±4.51 | 0.05 |
| HDL-c, mg/dL | 46.63±0.46 | 49.57±0.86 | 46.09±0.78 | 44.18±0.76 | 44.63±0.71 | < 0.0001 |
| Educational level, % |  |  |  |  |  | < 0.0001 |
| Below high school | 10.62(7.74,13.49) | 2.77(0.70, 4.85) | 6.58(2.44,10.72) | 15.02(11.64,18.40) | 23.92(18.69,29.14) |  |
| High school | 27.43(21.98,32.88) | 26.99(22.32,31.66) | 26.83(17.90,35.76) | 26.84(22.79,30.88) | 29.98(24.44,35.51) |  |
| Above high school | 61.95(54.07,69.84) | 70.24(64.94,75.54) | 66.59(57.95,75.23) | 58.15(53.23,63.06) | 46.11(39.83,52.38) |  |
| Marital status, % |  |  |  |  |  | 0.03 |
| Married or living with a partner | 81.21(68.91,93.51) | 83.14(78.84,87.45) | 73.68(67.36,80.00) | 82.43(79.45,85.42) | 80.31(76.03,84.59) |  |
| Living alone | 18.79(15.95,21.63) | 16.86(12.55,21.16) | 26.32(20.00,32.64) | 17.57(14.58,20.55) | 19.69(15.41,23.97) |  |
| PIR, % |  |  |  |  |  | < 0.0001 |
| PIR≤1.3 | 10.36(7.14,13.59) | 6.83(4.27, 9.38) | 9.49(5.34,13.65) | 14.37(8.63,20.10) | 12.05(9.05,15.05) |  |
| 1.3<PIR≤3.5 | 29.77(24.85,34.69) | 25.52(21.38,29.65) | 21.04(13.54,28.55) | 29.58(25.82,33.34) | 46.59(41.06,52.12) |  |
| PIR>3.5 | 59.86(51.21,68.52) | 67.66(62.68,72.63) | 69.46(61.48,77.45) | 56.05(48.75,63.35) | 41.37(34.55,48.18) |  |
| BMI category, % |  |  |  |  |  | 0.40 |
| <25 kg/m^2^ | 21.17(16.90,25.44) | 24.04(19.63,28.44) | 18.51(11.27,25.75) | 19.02(13.86,24.17) | 20.41(16.23,24.59) |  |
| 25-30 kg/m^2^ | 45.13(38.67,51.58) | 46.29(41.41,51.17) | 45.80(38.78,52.83) | 44.34(39.87,48.82) | 43.32(36.54,50.09) |  |
| >=30 kg/m^2^ | 33.71(28.39,39.03) | 29.67(24.12,35.23) | 35.69(27.75,43.62) | 36.64(29.53,43.74) | 36.27(30.82,41.72) |  |
| Age category, % |  |  |  |  |  | < 0.0001 |
| <60y | 70.15(61.11,79.18) | 99.81(99.43,100.19) | 97.42(95.16, 99.68) | 55.91(50.83, 60.99) | 6.34(1.65, 11.02) |  |
| ≥60y | 29.85(24.86,34.84) | 0.19(-0.19, 0.57) | 2.58(0.32, 4.84) | 44.09(39.01,49.17) | 93.66(88.98,98.35) |  |
| Alcohol intake, % |  |  |  |  |  | < 0.0001 |
| No | 27.04(20.39,33.68) | 20.95(16.16,25.74) | 21.28(13.09,29.47) | 29.31(21.57,37.04) | 41.42(32.91,49.94) |  |
| Yes | 72.96(62.82,83.11) | 79.05(74.26,83.84) | 78.72(70.53,86.91) | 70.69(62.96,78.43) | 58.58(50.06,67.09) |  |
| Smoking, % |  |  |  |  |  | < 0.0001 |
| Never | 38.55(33.08,44.02) | 54.82(48.94,60.71) | 32.64(24.80,40.49) | 26.05(21.38,30.72) | 27.85(22.66,33.04) |  |
| Former | 39.33(32.91,45.76) | 34.69(29.33,40.06) | 38.50(30.99,46.02) | 37.51(33.62,41.40) | 53.93(48.25,59.60) |  |
| Now | 22.11(17.76,26.47) | 10.48(7.00,13.97) | 28.86(22.07,35.64) | 36.43(31.76,41.11) | 18.22(12.09,24.36) |  |
| Vigorous activity, % |  |  |  |  |  | < 0.0001 |
| No | 66.23(56.84,75.62) | 52.40(46.83,57.97) | 62.67(52.21,73.14) | 75.41(69.99,80.83) | 84.44(79.98,88.90) |  |
| Yes | 33.77(27.86,39.68) | 47.60(42.03,53.17) | 37.33(26.86,47.79) | 24.59(19.17,30.01) | 15.56(11.10,20.02) |  |
| Moderate activity, % |  |  |  |  |  | 0.001 |
| No | 41.19(35.41,46.97) | 34.03(28.87,39.18) | 36.00(26.04,45.96) | 47.33(41.95,52.71) | 50.70(44.68,56.71) |  |
| Yes | 58.81(49.67,67.95) | 65.97(60.82,71.13) | 64.00(54.04,73.96) | 52.67(47.29,58.05) | 49.30(43.29,55.32) |  |
| History of DM, % |  |  |  |  |  | < 0.0001 |
| No | 87.03(75.32,98.73) | 98.85(97.50,100.21) | 92.17(86.90, 97.44) | 83.64(79.64, 87.65) | 61.88(56.19,67.57) |  |
| Yes | 12.97(9.97,15.97) | 1.15(-0.21, 2.50) | 7.83(2.56,13.10) | 16.36(12.35,20.36) | 38.12(32.43,43.81) |  |
| History of CVD, % |  |  |  |  |  | < 0.0001 |
| No | 92.24(79.88,104.61) | 97.35(96.17,98.53) | 95.85(92.97,98.73) | 89.79(86.74,92.83) | 82.10(77.19,87.01) |  |
| Yes | 7.76(5.53, 9.98) | 2.65(1.47, 3.83) | 4.15(1.27, 7.03) | 10.21(7.17,13.26) | 17.90(12.99,22.81) |  |
| History of hypertension, % |  |  |  |  |  | < 0.0001 |
| No | 56.62(47.63,65.62) | 74.06(68.07,80.06) | 64.76(54.66,74.85) | 46.78(41.60,51.96) | 27.76(22.46,33.06) |  |
| Yes | 43.38(36.32,50.43) | 25.94(19.94,31.93) | 35.24(25.15,45.34) | 53.22(48.04,58.40) | 72.24(66.94,77.54) |  |
| History of hyperlipidemia, % |  |  |  |  |  | < 0.001 |
| No | 17.57(14.10,21.04) | 26.21(21.46,30.95) | 14.42(5.72,23.13) | 10.19(6.96,13.42) | 13.22(9.31,17.13) |  |
| Yes | 82.43(71.15,93.71) | 73.79(69.05,78.54) | 85.58(76.87,94.28) | 89.81(86.58,93.04) | 86.78(82.87,90.69) |  |
| History of ED, % |  |  |  |  |  | < 0.0001 |
| No | 74.01(64.83,83.19) | 93.60(91.33,95.87) | 82.37(75.98,88.76) | 65.51(60.85,70.16) | 37.60(32.17,43.03) |  |
| Yes | 25.99(20.63,31.35) | 6.40(4.13, 8.67) | 17.63(11.24,24.02) | 34.49(29.84,39.15) | 62.40(56.97,67.83) |  |
| History of severe ED, % |  |  |  |  |  | < 0.0001 |
| No | 52.94(46.79,59.08) | 75.81(72.20,79.42) | 60.53(53.29,67.76) | 41.98(37.40,46.55) | 13.96(9.65,18.26) |  |
| Yes | 47.06(38.39,55.73) | 24.19(20.58,27.80) | 39.47(32.24,46.71) | 58.02(53.45,62.60) | 86.04(81.74,90.35) |  |

**Abbreviations:**

ED: erectile dysfunction; BMI: body mass index; PIR: poverty income ratio; TC: total cholesterol; HDL-c: high-density lipoprotein cholesterol; ASCVD: atherosclerotic cardiovascular disease; DM: diabetes mellitus; CVD: cardiovascular disease.
