# Supplementary material for: Association between erectile dysfunction and the predicted 10-year risk for atherosclerosis cardiovascular disease among U.S. men: a population-based study from the NHANES 2001-2004
Source: Front Endocrinol (Lausanne). 2024 Dec 17;15:1442904. doi: 10.3389/fendo.2024.1442904 (PMC11685050; doi:10.3389/fendo.2024.1442904)
Supplement: Supplementary file 3 [file Table2.docx]

**Table S2.** Multivariable logistic regression analyses for 10-year ASCVD risk score and ED, weighted.

| Exposure | Adjusted Model 1 | | Adjusted Model 2 | | Adjusted Model 3 | |
| --- | --- | --- | --- | --- | --- | --- |
|  | OR (95%CI) | P value | OR (95%CI) | P value | OR (95%CI) | P value |
| ASCVD risk score, continuous | 1.10(1.08, 1.12) | <0.0001 | 1.06(1.05,1.08) | <0.0001 | 1.04(1.02,1.06) | <0.001 |
| ASCVD risk score, category |  |  |  |  |  |  |
| <5% (Low) | Reference | Reference | Reference | Reference | Reference | Reference |
| 5%-7.5% (Borderline) | 3.13(1.77, 5.56) | <0.001 | 3.11(1.70, 5.70) | <0.001 | 2.95(1.60, 5.44) | 0.003 |
| 7.5%-20% (Intermediate) | 7.70(4.76, 12.47) | <0.0001 | 5.01(2.76, 9.08) | <0.0001 | 4.53(2.35, 8.73) | <0.001 |
| >20% (High) | 24.28(14.77, 39.93) | <0.0001 | 10.51(4.90,22.55) | <0.0001 | 7.62(3.19,18.19) | <0.001 |
| P for trend | <0.0001 |  | <0.0001 |  | <0.001 |  |

**Abbreviations:**

ED: erectile dysfunction; BMI: body mass index; PIR: poverty income ratio; ASCVD: atherosclerotic cardiovascular disease; DM: diabetes mellitus; CVD: cardiovascular disease; OR: odds ratios; 95%CI: 95% confidence intervals.

Model 1: unadjusted.

Model 2: adjusted for age, education level, marital status, and PIR.

Model 3: age, education level, marital status, PIR, BMI, hypertension, DM, CVD, hyperlipidemia, alcohol consumption, smoking status, vigorous activity, and moderate activity.
