# Supplementary material for: Association between erectile dysfunction and the predicted 10-year risk for atherosclerosis cardiovascular disease among U.S. men: a population-based study from the NHANES 2001-2004
Source: Front Endocrinol (Lausanne). 2024 Dec 17;15:1442904. doi: 10.3389/fendo.2024.1442904 (PMC11685050; doi:10.3389/fendo.2024.1442904)
Supplement: Supplementary file 4 [file Table3.docx]

**Table S3.** Sensitivity analysis for 10-year ASCVD risk score and ED, weighted.

| Exposure | Adjusted Model 1 | | Adjusted Model 2 | | Adjusted Model 3 | |
| --- | --- | --- | --- | --- | --- | --- |
|  | OR (95%CI) | P value | OR (95%CI) | P value | OR (95%CI) | P value |
| ASCVD risk score, continuous | 1.13(1.11,1.14) | <0.0001 | 1.09(1.06,1.12) | <0.0001 | 1.07(1.04,1.10) | <0.001 |
| ASCVD risk score, category |  |  |  |  |  |  |
| <5% (Low) | Reference | Reference | Reference | Reference | Reference | Reference |
| 5%-7.5% (Borderline) | 2.04(1.47, 2.84) | <0.001 | 2.01(1.43, 2.84) | <0.001 | 1.73(1.19,2.51) | 0.01 |
| 7.5%-20% (Intermediate) | 4.33(3.36, 5.58) | <0.0001 | 2.86(2.02, 4.06) | <0.0001 | 2.01(1.27,3.20) | 0.01 |
| >20% (High) | 19.32(12.58, 29.68) | <0.0001 | 7.60(4.07,14.20) | <0.0001 | 4.11(1.97,8.54) | 0.002 |
| P for trend | <0.0001 |  | <0.0001 |  | 0.001 |  |

**Abbreviations:**

ED: erectile dysfunction; BMI: body mass index; PIR: poverty income ratio; ASCVD: atherosclerotic cardiovascular disease; DM: diabetes mellitus; CVD: cardiovascular disease; OR: odds ratios; 95%CI: 95% confidence intervals.

Model 1: unadjusted.

Model 2: adjusted for age, education level, marital status, and PIR.

Model 3: age, education level, marital status, PIR, BMI, hypertension, DM, CVD, hyperlipidemia, alcohol consumption, smoking status, vigorous activity, and moderate activity.
